# Phylogenetic Identification of Fungi Isolated from the Marine Sponge *Tethya aurantium* and Identification of Their Secondary Metabolites

**DOI:** 10.3390/md9040561

**Published:** 2011-04-06

**Authors:** Jutta Wiese, Birgit Ohlendorf, Martina Blümel, Rolf Schmaljohann, Johannes F. Imhoff

**Affiliations:** Kieler Wirkstoff-Zentrum (KiWiZ) at the IFM-GEOMAR (Leibniz-Institute of Marine Sciences), Am Kiel-Kanal, 44, 24106, Kiel, Germany; E-Mails: jwiese@ifm-geomar.de (J.W.); bohlendorf@ifm-geomar.de (B.O.); mbluemel@ifm-geomar.de (M.B.); rschmaljohann@ifm-geomar.de (R.S.)

**Keywords:** *Tethya aurantium*, sponge-associated fungi, phylogenetic analysis, natural products, cillifuranone

## Abstract

Fungi associated with the marine sponge *Tethya aurantium* were isolated and identified by morphological criteria and phylogenetic analyses based on internal transcribed spacer (ITS) regions. They were evaluated with regard to their secondary metabolite profiles. Among the 81 isolates which were characterized, members of 21 genera were identified. Some genera like *Acremonium*, *Aspergillus*, *Fusarium*, *Penicillium*, *Phoma*, and *Trichoderma* are quite common, but we also isolated strains belonging to genera like *Botryosphaeria*, *Epicoccum*, *Parasphaeosphaeria*, and *Tritirachium* which have rarely been reported from sponges. Members affiliated to the genera *Bartalinia* and *Volutella* as well as to a presumably new *Phoma* species were first isolated from a sponge in this study. On the basis of their classification, strains were selected for analysis of their ability to produce natural products. In addition to a number of known compounds, several new natural products were identified. The scopularides and sorbifuranones have been described elsewhere. We have isolated four additional substances which have not been described so far. The new metabolite cillifuranone (**1**) was isolated from *Penicillium chrysogenum* strain LF066. The structure of cillifuranone (**1**) was elucidated based on 1D and 2D NMR analysis and turned out to be a previously postulated intermediate in sorbifuranone biosynthesis. Only minor antibiotic bioactivities of this compound were found so far.

## 1. Introduction

Natural products are of considerable importance in the discovery of new therapeutic agents [1]. Apart from plants, bacteria and fungi are the most important producers of such compounds [2]. For a long time neglected as a group of producers of natural products, marine microorganisms have more recently been isolated from a variety of marine habitats such as sea water, sediments, algae and different animals to discover new natural products [3,4]. In particular, sponges which are filter feeders and accumulate high numbers of microorganisms have attracted attention [5,6]. Though the focus of most of these investigations was concerned with the bacteria, a series of investigations identified marine sponges also as a good source of fungi [7–18]. Due to the accumulation of microorganisms, it is no surprise that sponges account for the majority of fungal species isolated from the marine realm [19]. However, the type of association and a presumable ecological function of accumulated fungi in sponges remain unclear and little evidence is available on fungi specifically adapted to live within sponges. One example is represented by fungi of the genus *Koralionastes*, which are known to form fruiting bodies only in close association with crustaceous sponges associated with corals [20].

Consistently, fungi isolated from sponges account for the highest number (28%) of novel compounds reported from marine isolates of fungi [19]. Marine isolates of fungi evidently are a rich source of chemically diverse natural products which has not been consequently exploited so far. Among a number of metabolites from sponge-associated fungi with promising biological activities are the cytotoxic gymnastatins and the p56^lck^ tyrosine kinase inhibitor ulocladol [21,11]. In view of these exciting data and our own previous work on the bacterial community associated with *Tethya aurantium* [22], we have now isolated and identified a larger number of fungi from this sponge. In European waters, *Tethya aurantium* is commonly found in the Atlantic Ocean, the English Channel, the North Sea as well as the Mediterranean Sea, where our specimens originated from [23]. Except for a single report from Indriani [24], fungi associated with *Tethya* sp. have not been investigated so far. The formation of natural products by these fungi and their biotechnological potential has not been evaluated yet.

For the identification of the fungal isolates from *Tethya aurantium*, we combined morphological criteria and phylogenetic analyses based on the sequence of the internal transcribed spacer (ITS) regions 1 and 2. On the basis of their classification, strains were selected for analysis of their ability to produce natural products. In addition to a number of known compounds, the new cyclodepsipeptides scopularide A and B were produced by a *Scopulariopsis brevicaulis* isolate [25]. Because of their antiproliferative activities against several tumor cell lines, these peptides and their activities have been patented [26]. During the present study, we have isolated four so far undescribed substances. Structure and properties of the new cillifuranone, a secondary metabolite from *Penicillium chrysogenum* strain LF066 are reported here.

## 2. Results and Discussion

### 2.1. Identification of the Fungal Strains Isolated from *T. aurantium*

In most studies on fungi associated with sponges the taxonomic classification of the fungi was based exclusively on morphological characteristics and in many cases identification was possible only at the genus level [17]. This can be attributed to the fact that taxonomic identification of fungi at the species level is not always easy. It is impaired by the fact that under laboratory conditions many fungi do not express reproductive features like conidia or ascomata, which represent important traits for identification. These fungi are classified as “mycelia sterilia”.

**Figure 1.**
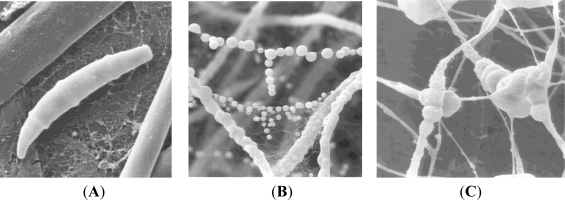
Scanning electron micrographs of *Fusarium* sp. strain LF236. (**A**) Multicellular, curved conidiospore; (**B**) Exudates in the surface layer of a liquid culture; (**C**) Intercalary chlamydospores in the mycelium.

Therefore, morphological criteria as well as sequence information and the determination of phylogenetic relationships are considered to be necessary for the identification of fungi. Consequently, we have combined the morphological characterization with a PCR-based analysis using ITS1-5.8S-rRNA-ITS2 gene sequences to identify 81 fungi isolated from *Tethya aurantium*. Based on these criteria the strains could be identified to the species level (Figure 1, Table 1).

First of all, morphological criteria enabled the identification of most of the fungal isolates at the genus level (Table 1). However, under the culture conditions applied, 11 strains did not produce spores and were designed as “mycelia sterilia”. A morphological classification of these strains was not possible. Despite the formation of spores, another four strains could not be classified on the basis of morphological characteristics.

**Figure 2.**
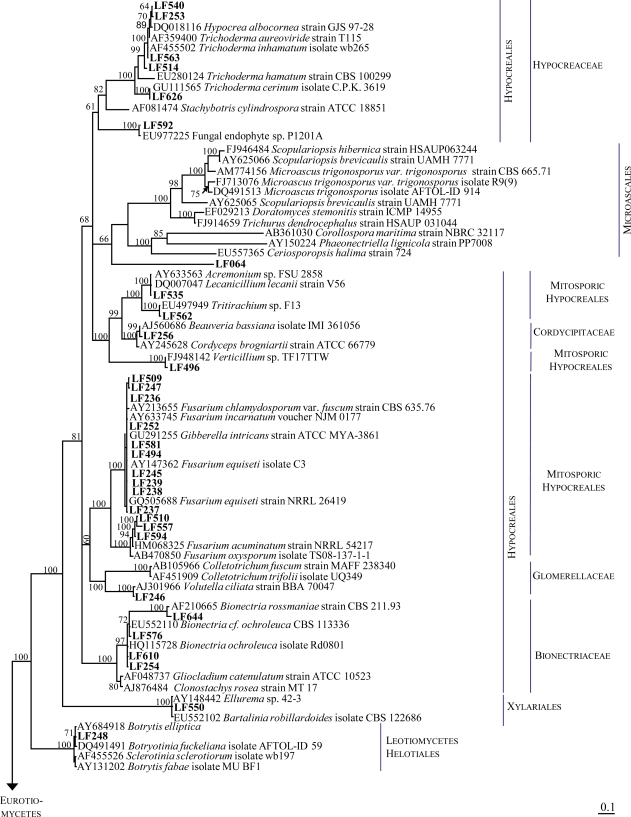

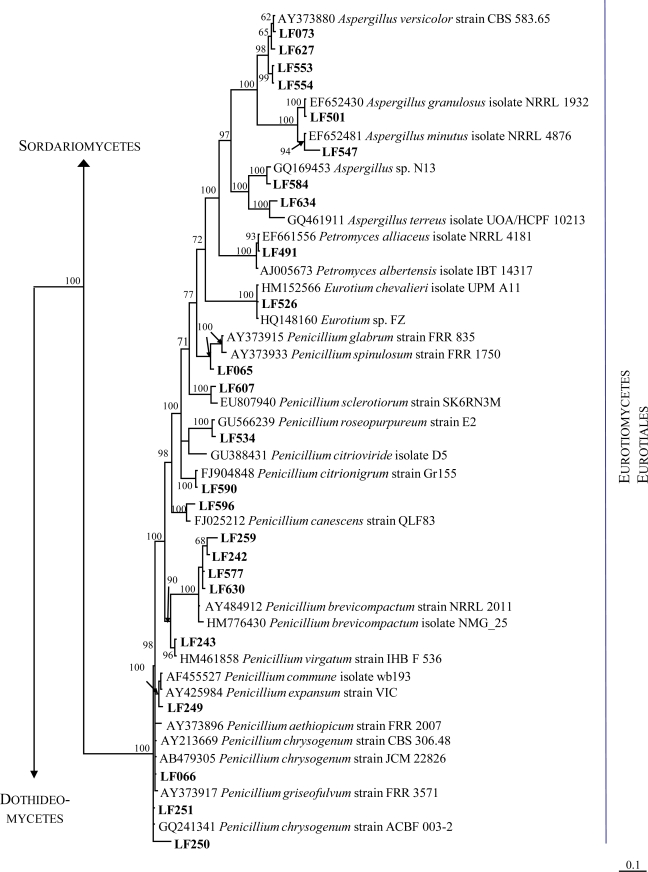

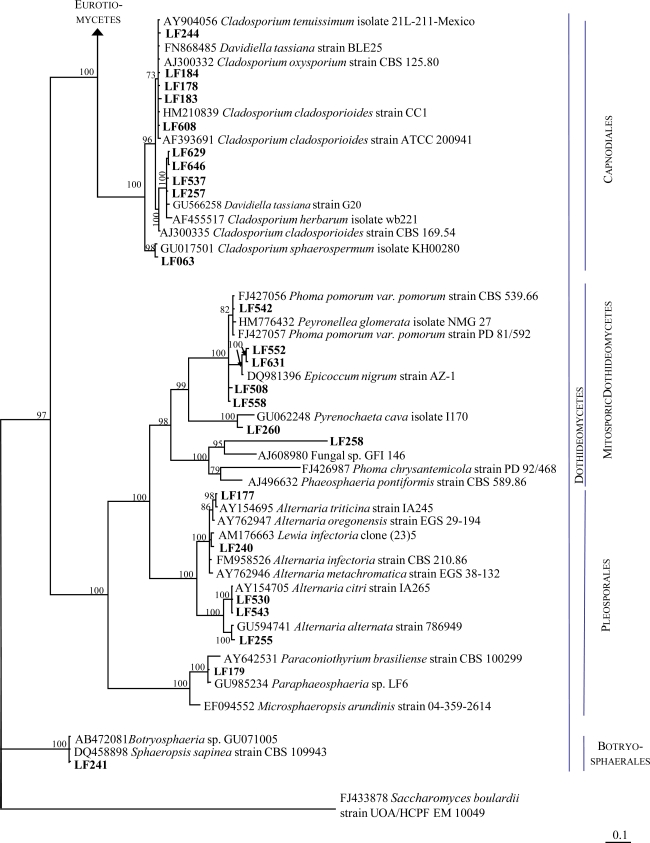
Phylogenetic consensus tree based on ITS1-5.8S-ITS2 gene sequences calculated by Bayesian inference assuming the general time reversible (GTR) model (6 substitution rate parameters, gamma-shaped rate variation, proportion of invariable sites). Isolates from *Tethya aurantium* obtained during this study are printed in bold. Numbers on nodes indicate Bayesian posterior probability values. nt = nucleotides.

In order to verify the results of the morphological examination and identify the strains at the species level, they were subjected to ITS1-5.8S-ITS2 gene sequence analysis. The results obtained from this sequence analysis corresponded well with those from the morphological identification (Table 1, Figure 2) and in addition allowed identification of those strains not identified microscopically. In most cases, the sequence data and the phylogenetic relationships allowed the identification at the species level. Results from BLAST search are depicted in Table 1. Taking together morphological and genetic characteristics, most isolates belong to the Ascomycotina with representatives of the fungal classes Dothideomycetes (24 isolates), Eurotiomycetes, (25 isolates), Sordariomycetes (30 isolates) and Leotiomycetes (1 isolate). Only a single isolate (strain LF538) was classified as belonging to the Mucoromycotina.

As shown in the phylogenetic tree (Figure 2), 10 isolates of the Dothideomycetes closely affiliated to fungal species of the order Capnodiales (*Cladosporium* spp. and its teleomorph *Davidiella*). Within the order Pleosporales (13 isolates) 5 isolates were closely related to *Alternaria* sp., including *Lewia infectoria* as teleomorph. From the same order we also isolated 2 strains of the species *Epicoccum nigrum* and 1 *Paraphaeosphaeria* strain. Furthermore, 5 isolates of the genus *Phoma* were found, of which one isolate (strain LF258) shows only 91% sequence similarity to *Septoria arundinacea* as next relative according to BLAST results (Table 1) and presumably represents a new species in the *Phoma* lineage. The low similarity of this isolate to known sequences is also reflected in the position in the phylogenetic tree, clustering distinct from other *Phoma* species. A single isolate (strain LF241) was assigned to *Sphaeropsis sapinea*/*Botryosphaeria* sp. within the order Botryosphaeriales in the class Dothideomycetes.

All 25 isolates assigned to the class Eurotiomycetes were affiliated to the order *Eurotiales* and are represented by 14 isolates closely affiliating to *Penicillium* species (*P. glabrum*, *P. virgatum*/ *P. brevicompactum*, *P. griseofulvum*/*P. commune*, *P. chrysogenum*, *P. sclerotiorum*/*P. citreonigrum*, *P. citreoviride*/*P. roseopurpureum*, *P. canescens*), 10 representatives of the genus *Aspergillus* (including 2 of its teleomorphs, *Petromyces alliaceus* and *Eurotium chevalieri*) and 1 isolate of *Paecilomyces.*

The 30 isolates affiliated to the class Sordariomycetes were grouped into the orders Hypocreales (26 isolates), Microascales (2 isolates) and Xylariales (1 isolate). Within the Hypocreales, one isolate each was closely related to *Beauveria bassiana* (strain LF256), *Tritirachium album* (strain LF562), *Verticillium* sp. (strain LF496), and *Volutella ciliata* (strain LF246). The most frequent genera within this order were *Fusarium* (13 isolates), *Trichoderma* (5 isolates, including *Hypocrea* teleomorphs) and *Clonostachys* (4 isolates, including teleomorphs of *Bionectria*). The Microascales were represented by two isolates of the genus *Scopulariopsis*. One of these (strain LF064), morphologically classified as *Scopulariopsis murina*, was distantly related to the next cultured relative (89% according to BLAST search) and appears as a sister group to the *Scopulariopsis* lineage in the phylogenetic tree. The order *Xylariales* was represented by only a single isolate (strain LF550), *Bartalinia robillardoides.* As the only representatives of the order Helotiales within the class Leotiomycetes strain LF248 was closely related to *Botryotinia fuckeliana*.

The combination of microscopic and genetic analyses has proven to be a reliable method for the identification of fungal isolates and results from both approaches corresponded quite well (Table 1, Figure 2). The reliable identification of isolates is a fundamental prerequisite in order to characterize the producers of marine natural products [3], to determine the occurrence of fungal species in different habitats, and to correlate distinct secondary metabolite patterns to fungal species. Therefore, we highly recommend that morphological identification of fungal isolates consequently should be verified by molecular means and thus raise the number of reliably identified species in public databases.

A number of investigations, mainly with the aim of finding novel natural products, showed that most marine sponges harbor a plethora of cultivable fungi within their tissue [10–15,18]. Despite the large number of fungi isolated from sponges, a selective accumulation of specific taxa within sponges and a truly marine nature of these fungi is doubted, which is why they are commonly referred to as “marine-derived” [27,28]. In fact, it could be shown that the phylogenetic diversity of fungi isolated from different sponges varies [17]. It has also been stated that the taxa frequently isolated from sponges resemble those described from terrestrial habitats [11,16,28]. This is in good accordance with our results, showing representatives of *Acremonium*, *Aspergillus*, *Fusarium*, *Penicillium*, *Phoma*, and *Trichoderma* to be abundant in *Tethya aurantium* and with those of Wang (2006) demonstrating that they also are widely distributed among different sponges from various locations [29]. Although it appears that the marine environment indeed provides various habitats for fungi, this has rarely been demonstrated. Nonetheless, due to the accumulation of fungi within sponges a large number of strains can be isolated which increases the probability to find representatives of less common taxa which might produce unprecedented secondary metabolites. For example, fungi belonging to the genera *Beauveria*, *Botryosphaeria*, *Epicoccum*, *Tritirachium*, and *Paraphaeosphaeria* have rarely been obtained from marine sponges [11,18,24] and, to the best of our knowledge, we have isolated *Bartalinia* sp. and *Volutella* sp. from a marine sponge for the first time.

Although evidence is presented that some bacterial symbionts of sponges are the producers of metabolites originally assumed to be produced by the sponge [4], equivalent evidence for fungi is lacking. There is actually little evidence for sponge-specific fungal associations and the only reports on this matter deals with the above mentioned *Koralionastes* species and a yeast living in symbiosis with the sponge *Chondrilla* sp. [30]. In fact, most of the studies had the biotechnological potential of sponge-derived fungi in mind but not the ecological role. Our culture-dependent approach was not considered to approach the aspect of specificity of the association with the sponge and molecular-based studies would be more suited to identify specifically sponge-associated fungi.

### 2.2. Secondary Metabolite Analyses

With the cultivation-based approach used in this study, we obtained a variety of strains from a surprisingly broad range of phylogenetic groups of fungi. A selection of the isolated and identified fungi was subjected to analysis of their secondary metabolite profiles. Strains were selected in order to represent a wide spectrum of genera, representatives of a variety of different genera, some known to include strains and species known for the production of secondary metabolites others from less common taxa. Some of the strains selected according to systematic criteria did not produce detectable amounts of secondary metabolites under the applied culture conditions. Unraveling their potential of secondary metabolite production will require more intense studies. Those extracts which did contain at least one compound in significant amounts were analyzed by HPLC with DAD (UV)- and MS-detection and the metabolites which could be identified are listed in Table 2. A high percentage of the substances identified this way could be verified by ^1^H NMR spectroscopy (see Table 2). The majority of these metabolites have been reported from fungi before. Two of our previous reports on metabolites from fungi isolated from *Tethya aurantium* deal with the antiproliferative scopularides A–B [25,26] and the sorbifuranones A–C [31].

For four compounds, database searches [33,42–43] did not lead to a hit (B, C, D) or no publication was available (A). The structure elucidations of compounds A and B, metabolites with modified diketopiperazine substructures, and compound D, a new azaphilone derivative, are in progress. Compound C was identified as the new metabolite cillifuranone and its structure is described in the following.

*Penicillium* strain LF066, the producer of cillifuranone, was singled out for further investigations, because first surveys proved it to be a very potent producer of secondary metabolites. From the same strain, sorbifuranone B and C as well as 2′,3′-dihydrosorbicillin have already been described by Bringmann *et al.* [31] and it was obvious that the full potential of the strain had not been exploited, yet. Further analysis led to the detection of xanthocillines and sorbifuranones and the isolation of sorbifuranone B, meleagrin, roquefortin C, a couple of ergochromes as well as the new cillifuranone (**1**) whose structure was elucidated based on 1D and 2D NMR experiments.

**Figure 3.**
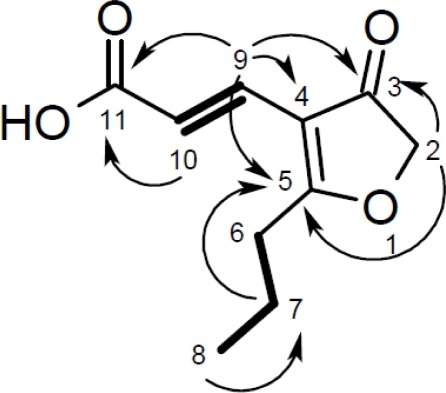
Spin systems deduced from the ^1^H-^1^H COSY spectrum (bold) and selected ^1^H-^13^C HBMC correlations (arrows) relevant to the structure elucidation of cillifuranone (**1**).

The ^13^C NMR spectrum of **1** displayed 10 clearly distinguishable carbon signals which was in good agreement with the molecular formula C_10_H_12_O_4_, deduced from the result of a HRESI-MS measurement (calculated for C_10_H_12_O_4_Na 219.0628, measured 219.0627). The carbon signals included resonances belonging to three carbonyl or enol carbons (δ_C_ 170.9, 196.7 and 201.7), three sp^3^ hybridized methylene carbons (δ_C_ 20.9, 31.6 and 76.3), one methyl group (δ_C_ 14.0), two olefinic methines (δ_C_ 118.5 and 133.0) and finally one quaternary olefinic carbon (δ_C_ 112.6). The structure of the molecule could be delineated from 1D (^1^H, ^13^C and DEPT) and 2D NMR (^1^H-^13^C HSQC, ^1^H-^1^H COSY and ^1^H-^13^C HMBC) spectra. From the ^1^H-^1^H COSY spectrum two separate spin systems could be identified. The first one consisted of the olefinic methine groups CH-9 (δ_C_ 133.0, δ_H_ 7.32) and CH-10 (δ_C_ 118.5, δ_H_ 6.83), forming an *E*-configured double bond as proven by their ^3^*J* coupling constant of 16 Hz. The corresponding protons H-9 and H-10 both showed ^1^H-^13^C HMBC correlations to the carboxyl carbon C-11 (δ_C_ 170.9) as well as to the quarternary carbons of the furanone ring, C-3 (δ_C_ 201.7) to C-5 (δ_C_ 196.7). C-3 was the ketone carbonyl group included in the furanone substructure which was in accordance with its chemical shift. C-4 (δ_C_ 112.6) and C-5 were also part of the furanone and formed a tetrasubstituted double bond in which C-5 was located adjacent to an oxygen atom. Compared to an unsubstituted enol, the resonance of C-5 was shifted further downfield due to the conjugation of the double bond Δ^4,5^ with the carbonyl carbon C-3. Apart from C-9 of the double bond Δ^9,10^, the carbonyl carbon C-3 and the oxygen atom of the furanone ring, Δ^4,5^ was also connected to C-6 of the second spin system consisting of the methylene groups CH_2_-6 (δ_C_ 31.6, δ_H_ 2.76) and CH_2_-7 (δ_C_ 20.9, δ_H_ 1.76) as well as the methyl-group CH_3_-8 (δ_C_ 14.0, δ_H_ 1.03). Thus, the second spin system evidently was an n-propyl-chain. The furanone ring was completed with the methylene group CH_2_-2 (δ_C_ 76.3, δ_H_ 4.67). Its ^1^H and ^13^C shifts proved it to be linked to an oxyen atom, the ^1^H-^13^C HMBC correlations to C-3 and C-5 secured its exact position. Thus, the structure of cillifuranone (**1**) could be unambiguously determined (Figure 3, Table 3).

Cillifuranone (**1**) was tested in a panel of bioassays evaluating the compound with respect to cytotoxic, antimicrobial and enzyme inhibitory activity. Very low activity was only found against *Xanthomonas campestris* (24% growth inhibition) and *Septoria tritici* (20% growth inhibition) at a concentration of 100 μM.

**Figure 4.**
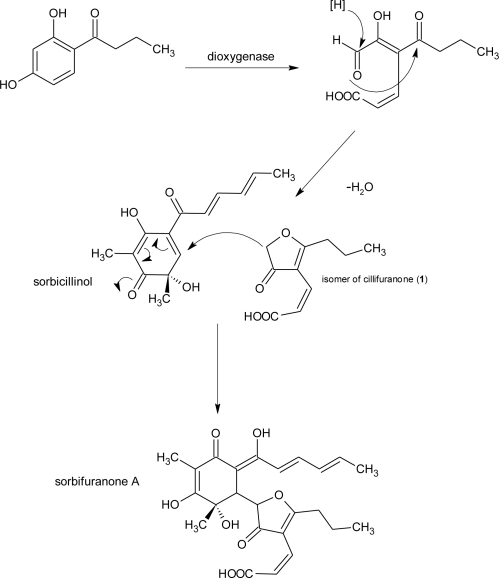
Biosynthesis of sorbifuranone A via Michael reaction of an isomer of cillifuranone (**1**) and sorbicillinol as postulated by Bringmann *et al.* (modified from [31]).

Strain LF066 was identified as *Penicillium chrysogenum*, a species that in our experience often produces metabolites deriving from sorbicillinol as a biosynthetic precursor (sorbicillinoids). The detection of bisvertinolone and the sorbifuranones in culture extracts of the fungus was consistent with this experience. Furanone substructures are abundant in natural products and can be found in metabolites from bacteria, fungi and plants [42] and presumably are products from different biosynthetic pathways [44–46]. From the genus *Penicillium* a number of furanone containing compounds has been described, including simple small molecules like penicillic acid, but also more complex ring structures such as the rotiorinols [47] or rugulovasines [48]. In most cases the furanone ring is a furan-2(5*H*)-one, whereas in cillifuranone (**1**) we have a furan-3(2*H*)-one. With that differentiation being made the number of related compounds gets fewer, furan-3(2*H*)-ones do not seem to be as ubiquituous as the furan-2(5*H*)-ones. From *Penicillium* strains, apart from the above mentioned sorbifuranones, berkeleyamide D [49] and trachyspic acid [50], both spiro compounds like sorbifuranone C, are examples. The new cillifuranone represents a substructure of the sorbifuranones, albeit with a different configuration of the exocyclic double bond, and represents the substructure which makes the sorbifuranones unique in the compound class of the sorbicillinoids. Just recently Bringmann *et al.* [31] published the structures of the sorbifuranones and postulated **1** to be an intermediate in their biosynthesis (Figure 4) which makes the isolation of **1** a very interesting result. The only difference between the postulated intermediate and our structure is, as stated above, the configuration of the double bond. However, in the crude extract of the strain, we detected two isomers with the same molecular weight and a very similar UV-spectrum, so that we assume that both isomers were present, but after the isolation process only the *E*-isomer was obtained, so that it might be the favoured configuration under the applied conditions.

## 3. Experimental Section

### 3.1. Sampling Sites

The Limsky kanal (Canal di Lemme or Limsky channel) is a semi-closed fjord-like bay in the Adriatic Sea nearby Rovinj (Istrian Peninsula, Croatia). It is situated along an east-west axis, with an approximate length of 1 km and a maximum width of about 650 m and reaches a maximum depth of 32 m [51]. The sampling site was located at N45°7972′ and E13°43,734′.

### 3.2. Sponge Collection

Several specimens (13) of the Mediterranean sponge *Tethya aurantium* were collected by scuba diving. They were obtained in April 2003, June 2004, May 2005 and August 2006 in a depth of 5–15 m. The sponges were collected into sterile plastic bags, cooled on ice and transported immediately to the laboratory, where they were washed three times with sterile filtered seawater (0.2 μm). The sponge tissue was then cut into small pieces of approximately 0.1 cm^3^ each, which were either placed directly onto GPY agar plates (LF236 to LF255) or homogenized and diluted with membrane-filtered seawater (all other isolates).

### 3.3. Isolation, Cultivation and Storage of Fungi

Fungi were isolated on a low nutrient GPY agar, based on natural seawater of 30 PSU, containing 0.1% glucose, 0.05% peptone, 0.01% yeast extract and 1.5% agar. Small pieces of sponge tissue or 50 μL of the homogenate (undiluted or 1:10 or 1:100 diluted with sterile seawater) were used as inoculum. The agar plates were incubated for periods of 3 days to 4 weeks and were checked regularly for fungal colonies, which were then transferred to GPY agar plates. Pure cultures were used for morphological identification by light microscopy and for scanning electron microscopy. Fungal isolates were stored as agar slant cultures at 5 °C, and additionally were conserved at −80 °C using Cryobank vials (Mast Diagnostica).

### 3.4. Morphological Identification of Fungal Isolates

The morphology of GPY agar grown fungal isolates was studied using a stereo microscope (10–80× magnification) and a phase-contrast microscope (300–500× magnification). By this method, the majority of spore-producing isolates could be identified up to the generic level using the tables of Barnett and Hunter [52] and, for more detailed descriptions, the site of MycoBank [53]. Morphological identification of the selected pure cultures was supported by light microscopy and scanning electron microscopy.

### 3.5. Scanning Electron Microscopy

For electron microscopy, young GPY agar colonies were cut in 1 cm^2^ samples, transferred through an ethanol series (30, 50, 70, 90, 3 × 100%; each 15 min) and subsequently critical-point-dried in liquid carbon dioxide (Balzers CPD030). Samples were sputter-coated with gold-palladium (Balzers SCD004) and analyzed with a ZEISS DSM 940 scanning electron microscope.

### 3.6. Genetic Identification of Fungal Isolates and Phylogenetic Analysis

DNA-extraction was performed using the Precellys 24 system (Bertin Technologies). To one vial of a Precellys grinding kit with a glass beads matrix (diameter 0.5 mm, peqlab Biotechnologies GmbH) 400 μL DNAse-free water (Fluka) were added. Cell material from the fungal culture was then transferred to this vial and homogenized two times for 45 s at a shaker frequency of 6500. The suspension was centrifuged at 6000 g for 10 min and 15 °C. The supernatant was stored at −20 °C until further use in PCR.

Fungal specific PCR by amplifying the ITS1-5.8S rRNA-ITS2 fragment was carried out using puReTaq™ Ready-To-Go™ PCR Beads (GE HEalthcare) with the ITS1 (5′-TCC GTA GGT GAA CCT GCG G-3′) and ITS4 (5′-TCC TCC GCT TAT TGA TAT GC-3′) primers according to White *et al.* [54]. PCR was conducted as follows: initial denaturation (2 min at 94 °C), 30 cycles of primer denaturation (40 s at 94 °C), annealing (40 s at 55 °C), and elongation (1 min at 72 °C) followed by a final elongation step (10 min at 72 °C). PCR products were checked for correct length (complete ITS1, 5.8S rRNA and ITS2 fragment length of *Penicillium brevicompactum* strain SCCM 10-I3 (EMBL-acc. No. EU587339 is 494 nucleotides), a 1% agarose gel in 1× TBE buffer (8.9 mM Tris, 8.9 mM borate, 0.2 mM EDTA).

PCR products were sequenced using the ABI PRISM^®^ BigDye™ Terminator Ready Reaction Kit (Applied Biosystems) on an ABI PRISM^®^ 310 Genetic Analyzer (Perkin Elmer Applied Biosystems). The ITS1 primer was used for sequencing. Sequence data were edited with ChromasPro Version 1.15 (Technelysium Pty Ltd.). Sequences from fungal strains obtained during this study were submitted to the EMBL database and were assigned accession numbers (FR822769-FR822849). Closest relatives were identified by sequence comparison with the NCBI Genbank database using BLAST (Basic Local Alignment Search Tool) [55]. Sequences were aligned using the ClustalX version 2.0 software [56] and the alignment was refined manually using BioEdit (version 7.0.9.0) [57]. For alignment construction, ITS1-5.8S-ITS2 gene fragments from closest cultured relatives according to BLAST as well as type strains were used, whenever possible. However, not from all fungal species, ITS sequences from type strains were available in NCBI/Genbank. The ITS1-5.8S-ITS2 gene sequence of *Saccharomyces boulardii* strain UOA/HCPF EM10049 (acc. No. FJ433878) was used as outgroup sequence for phylogenetic calculations. Phylogenetic calculations were performed with all closest relatives according to BLAST results (data not shown). For clarity, not all of these sequences were included in Figure 2. Phylogeny was inferred using MrBayes version 3.1 [58,59], assuming the GTR (general time reversible) phylogenetic model with 6 substitution rate parameters, a gamma-shaped rate variation with a proportion of invariable sites and default priors of the program. 1,000,000 generations were calculated and sampled every 1000th generation. Burn-in frequency was set to 25% of the samples. The consensus tree was edited in Treeview 1.3 [60].

### 3.7. Fermentation and Production of Extracts

The fungi were inoculated in 2 L Erlenmeyer flasks containing 750 mL modified Wickerham-medium [61], which consisted of 1% glucose, 0.5% peptone, 0.3% yeast extract, 0.3% malt extract, 3% sodium chloride (pH = 6.8). After incubation for 11–20 days at 28 °C in the dark as static cultures, extracts of the fungi were obtained. The mycelium was separated from the culture medium and extracted with ethanol (150 mL). The fermentation broth was extracted with ethyl acetate (400 mL). Both extracts were combined. Alternatively, cells and mycelium were not separated and the culture broth was extracted together with the cells of some cultures using ethyl acetate. After evaporation of the solvents, the powdery residue was reextracted with ethyl acetate (100 mL). The resulting residues were dissolved in 20 mL methanol and subjected to analytical HPLC-DAD-MS.

### 3.8. Chemical Analysis

UV-spectra of the identified metabolites were obtained on a NanoVue (GE Healthcare). NMR spectra were recorded on a Bruker DRX500 spectrometer (500 and 125 MHz for ^1^H and ^13^C NMR, respectively), using the signals of the residual solvent protons and the solvent carbons as internal references (δ_H_ 3.31 ppm and δ_C_ 49.0 ppm for methanol-*d*4). High-resolution mass spectra were acquired on a benchtop time-of-flight spectrometer (MicrOTOF, Bruker Daltonics) with positive electrospray ionization. Analytical reversed phase HPLC-DAD-MS experiments were performed using a C_18_ column (Phenomenex Onyx Monolithic C18, 100 × 3.00 mm) applying an H_2_O (A)/MeCN (B) gradient with 0.1% HCOOH added to both solvents (gradient: 0 min 5% B, 4 min 60% B, 6 min 100% B; flow 2 mL/min) on a VWR Hitachi Elite LaChrom system coupled to an ESI-ion trap detector (Esquire 4000, Bruker Daltonics).

Preparative HPLC was carried out using a VWR system consisting of a P110 pump, a P311 UV detector, a smartline 3900 autosampler and a Phenomenex Gemini-NX C18 110A, 100 × 50 mm, column or a Merck Hitachi system consisting of an L-7150 pump, an L-2200 autosampler and an L-2450 diode array detector and a Phenomenex Gemini C18 110A AXIA, 100 × 21.20 mm, column.

For the preparation of cillifuranone (**1**), the same solvents were used as for the analytical HPLC, with a gradient from 10% B, increasing to 60% B in 17 min, to 100% B from 17 to 22 min. **1** eluted with a retention time of 6.8 min and the amount of 23.2 mg of **1** could be obtained from a culture volume of 10 L.

Properties of cillifuranone (**1**): pale yellow needles; UV (MeOH) λ_max_ (logɛ) 278 (4.36); for 1D and 2D NMR data see Table 3 and SI; HRESIMS *m/z* 219.0627 (calcd for C_10_H_12_O_4_Na 219.0628).

### 3.9. Bioassays

Possible antimicrobial effects of cillifuranone (100 μM) were tested against *Bacillus subtilis* (DSM 347), *Brevibacterium epidermidis* (DSM 20660), *Dermabacter hominis* (DSM 7083), *Erwinia amylovora* (DSM 50901), *Escherichia coli* K12 (DSM 498), *Pseudomonas fluorescens* (NCIMB 10586), *Propionibacterium acnes* (DSM 1897^T^), *Pseudomonas aeruginosa* (DSM 50071), *Pseudomonas syringae* pv. *aptata* (DSM 50252), *Staphylococcus epidermidis* (DSM 20044), *Staphylococcus lentus* (DSM 6672), *Xanthomonas campestris* (DSM 2405), *Candida albicans* (DSM 1386), *Phytophthora infestans*, and *Septoria tritici* according to Schneemann *et al.* [62]. In addition, cytotoxic activity of cillifuranone (50 μM) towards the human hepatocellular carcinoma cell line HepG2 and the human colon adenocarcinom cell line HT29 were performed according to Schneemann *et al.* [62]. Inhibitory activities of cillifuranone (10 µM) against the enzymes acetylcholinesterase, phosphodiesterase (PDE-4B2), glycogen synthase kinase 3β, protein tyrosin phosphatase 1B, and HIV-1 reverse transcriptase were tested according to Helaly *et al.* [63].

## 4. Conclusion

The marine sponge *T. aurantium* was found to be a valuable source of secondary metabolite producing fungi. In addition to a variety of known substances, several new natural products were found and it is likely that additional ones can be identified during further studies. The antiproliferative active scopularides [25,26] were the first new metabolites from a *Scopulariopsis* species isolated from *T. aurantium*. The new cillifuranone (**1**) is a second example of a natural product produced by a fungal isolate from *T. aurantium*. Additional compounds were detected of which the chemical structures are not yet described. The application of alternative cultivation methods, which have not been used so far, are expected to further increase the spectrum of produced metabolites of our isolates obtained from *T. aurantium*.

The combination of morphological criteria and the results of the ITS1-5.8S-ITS2 fragment sequencing have been proven to be a valuable tool for the identification of fungal isolates. Apart from representatives of genera, which are widely distributed in terrestrial samples and in addition also reported from different sponges, we also identified members of taxa which so far have not been described to be associated with sponges. These strains distantly affiliated to *Bartalinia* sp. and *Votutella* sp. and one strain most likely is a new *Phoma* species.

## Figures and Tables

**Table 1.** Identification of fungal strains isolated from *Tethya aurantium* samples based on morphological criteria as well as genetic analysis of the internal transcribed spacer (ITS) region. Closest relatives to fungal strains according to BLAST search are presented. In case BLAST search yielded a cultured but undesignated strain as closest relative, the closest cultured and the designated relative is given additionally.

**Strain**	**Morphological identification**	**Seq. length (nt)**	**Next related cultivated strain (BLAST)**	**Acc. No.**	**Similarity (%)**	**Overlap (nt)**
LF063	*Cladosporium* sp.	483	Fungal sp. ARIZ AZ0920 *Cladosporium sphaerospermum* isolate KH00280	HM123596.1 GU017501.1	99 99	482 482
LF064	*Scopulariopsis murina*	473	Ascomycota sp. 840 *Phialemonium obovatum* strain CBS 279.76	GU934604.1 AB278187.1	92 89	360 340
LF065	*Penicillium* sp.	546	*Penicillium glabrum* strain 4AC2K	GU372904.1	99	545
LF066	*Penicillium* sp.	551	*Penicillium chrysogenum* strain JCM 22826	AB479305.1	99	549
LF073	*Aspergillus* sp.	533	*Aspergillus versicolor* isolate UOA/HCPF 8709	FJ878627.1	100	532
LF177	*Alternaria* sp.	568	*Lewia infectoria* strain IA310	AY154718	99	561
LF178	*Cladosporium* sp.	479	Fungal endophyte sp. g6 *Cladosporium cladosporioides* strain CC1	HM537022.1 HM210839.1	100 100	479 479
LF179	Mycelia sterilia	559	Fungal endophyte isolate 9137 *Paraphaeosphaeria* sp. LF6	EF419991.1 GU985234.1	100 99	555 557
LF183	*Cladosporium* sp.	523	*Dothideomycetes* sp. 11366 *Cladosporium cladosporioides* isolate SLP001	GQ153254.1 FJ932747.1	99 99	522 521
LF184	*Cladosporium* sp.	475	Fungal endophyte sp. g6 *Cladosporium cladosporioides* strain CC1	HM537022.1 HM210839.1	100 100	475 475
LF236	*Fusarium* sp.	512	*Fusarium* sp. CPK3469 *Gibberella intricans* strain ATCC MYA-3861	FJ827615.1 GU291255.1	99 99	511 511
LF237	*Fusarium* sp.	503	*Fusarium* sp*. CPK3337* *Fusarium equiseti* strain *NRRL 36478*	FJ827616.1 GQ505743.1	100 100	503 503
LF238	*Fusarium* sp.	513	*Fusarium* sp. CPK3469 *Fusarium equiseti* strain NRRL 36478	FJ827615.1 GQ505743.1	100 100	513 513
LF239	*Fusarium* sp.	509	*Fusarium* sp. NRRL 45997 *Fusarium equiseti* strain NRRL 36478	GQ505761.1 GQ505743.1	99 99	503 503
LF240	Mycelia sterilia	528	*Lewia* sp. B32C *Lewia infectoria* strain IA241	EF432279.1 AY154692.1	99 99	525 525
LF241	Mycelia sterilia	513	*Botryosphaeria* sp. GU071005 *Sphaeropsis sapinea* strain CBS109943	AB472081.1 DQ458898.1	100 100	512 512
LF242	*Penicillium* sp.	538	*Penicillium brevicompactum* isolate H66s1	EF634441.1	99	537
LF243	*Penicillium* sp.	532	*Penicillium virgatum* strain IHB F 536	HM461858.1	100	530
LF244	*Cladosporium* sp.	516	Fungal endophyte sp. g2 *Davidiella tassiana* strain BLE25	HM537019.1 FN868485.1	99 99	516 516
LF245	*Fusarium* sp.	494	*Fusarium* sp. CPK3514 *Fusarium equiseti* strain NRRL 13402	FJ840530.1 GQ505681.1	100 100	494 494
LF246	*Volutella* sp.	548	*Volutella ciliata* strain BBA 70047	AJ301966.1	99	547
LF247	*Fusarium* sp.	520	*Fusarium* sp. LD-135 *Fusarium equiseti* strain NRRL 13402	EU336989.1 GQ505681.1	99 99	509 504
LF248	*Botrytis* sp.	504	Fungal endophyte sp. g18 *Botryotinia fuckeliana* strain OnionBC-1	HM537028.1 FJ169667.2	100 100	504 504
LF249	*Penicillium* sp.	552	*Penicillium* sp. BM *Penicillium commune* isolate HF1	GU566211.1 GU183165.1	99 99	551 551
LF250	*Penicillium* sp.	564	*Penicillium chrysogenu*m strain ACBF 003-2	GQ241341.1	97	547
LF251	*Penicillium* sp	550	*Penicillium* sp. F6 *Penicillium chrysogenum* strain ACBF 003-2	GU566250.1 GQ241341.1	100 100	550 550
LF252	*Fusarium* sp	513	*Fusarium* sp. NRRL 45997 *Fusarium equiseti* strain NRRL 36478	GQ505761.1 GQ505743.1	99 99	511 511
LF253	*Trichoderma* sp.	543	*Hypocrea lixii* strain OY3207	FJ571487.1	100	540
LF254	*Clonostachys* sp	525	*Bionectria ochroleuca* strain G11	GU566253.1	100	524
LF255	*Alternaria* sp	518	Fungal endophyte sp. g76 *Alternaria alternata* strain 786949	HM537053.1 GU594741.1	100 100	518 518
LF256	*Botrytis* sp.	535	*Beauveria bassiana* strain G61	GU566276.1	99	533
LF257	*Cladosporium* sp.	495	*Davidiella tassiana* strain G20	GU566258.1	100	495
LF258	*Phoma* sp. nov.	538	Fungal sp. GFI 146 *Septoria arundinacea* isolate BJDC06	AJ608980.1 GU361965.1	93 91	470 460
LF259	*Penicillium* sp.	522	*Penicillium brevicompactum* strain: JCM 22849	AB479306.1	100	522
LF260	Sphaeropsidales	508	*Pyrenochaeta cava* isolate olrim63	AY354263.1	100	508
LF491	*Aspergillus* sp.	555	*Petromyces alliaceus* isolate NRRL 4181	EF661556.1	99	555
LF494	*Fusarium* sp.	469	*Fusarium* sp. CB-3	GU932675.1	100	469
LF496	Mycelia sterilia	531	*Verticillium* sp. TF17TTW	FJ948142.1	99	529
LF501	*Aspergillus* sp.	514	*Aspergillus granulosus* isolate NRRL 1932	EF652430.1	100	514
LF508	Not identified	501	*Phoma* sp. W21	GU045305.1	99	497
LF509	*Fusarium* sp.	504	*Fusarium* sp. CPK3514	FJ840530.1	99	503
LF510	*Fusarium* sp.	516	*Fusarium* sp. FL-2010c isolate UASWS0396	HQ166535.1	99	514
LF514	*Trichoderma* sp.	546	*Trichoderma* sp. TM9	AB369508.1	100	546
LF526	*Eurotium* sp.	486	*Eurotium* sp. FZ *Eurotium chevalieri* isolate UPM A11	HQ148160.1 HM152566.1	100 100	486 486
LF530	*Alternaria* sp.	522	*Alternaria* sp. 7 HF-2010	HQ380788.1	100	522
LF534	*Penicillium* sp.	540	*Penicillium roseopurpureum* strain E2	GU566239.1	99	536
LF535	*Acremonium* sp.	525	*Acremonium* sp. FSU2858 *Lecanicillium lecanii* strain V56	AY633563.1 DQ007047.1	99 99	523 510
LF537	*Cladosporium* sp.	503	*Cladosporium cladosporioides* strain F12	HQ380766.1	100	503
LF538	*Mucor hiernalis*	598	*Mucor hiemalis* isolate UASWS0442	HQ166553.1	100	598
LF540	Not identified	578	*Hypocrea lixii* isolate FZ1302	HQ259308.1	99	575
LF542	Mycelia sterilia	458	*Peyronellaea glomerata* isolate NMG_27 *Phoma pomorum* var. *pomorum* strain CBS 539.66	HM776432 FJ427056.1	99 99	457 457
LF543	*Alternaria* sp.	522	*Alternaria citri* strain IA265	AY154705.1	100	522
LF547	*Aspergillus* sp.	539	*Aspergillus minutus* isolate NRRL 4876	EF652481.1	98	529
LF550	Mycelia sterilia	524	*Bartalinia robillardoides* CBS:122686 *Ellurema* sp. 42-3	EU552102.1 AY148442.1	99 99	522 514
LF552	*Epicoccum nigrum*	506	*Epicoccum nigrum* strain GrS7	FJ904918.1	99	503
LF553	*Aspergillus* sp.	528	*Aspergillus* sp. Da91	HM991178.1	100	528
LF554	*Aspergillus* sp.	525	*Aspergillus* sp. Da91	HM991178.1	100	525
LF557	Mycelia sterilia	528	*Fusarium* sp. FL-2010f *Fusarium oxysporum* strain TS08-137-1-1	HQ166539.1 AB470850.1	99	522
LF558	Not identified	498	*Phoma* sp. W21	GU045305.1	100	498
LF562	*Tritirachium* sp.	504	*Tritirachium* sp. F13	EU497949.1	99	498
LF563	*Trichoderma* sp.	537	*Hypocrea lixii* isolate DLEN2008014	HQ149778.1	100	537
LF576	*Clonostachys* sp.	504	*Bionectria* cf. *ochroleuca* CBS 113336	EU552110.1	99	503
LF577	*Penicillium* sp.	538	*Penicillium brevicompactum* isolate NMG_25	HM776430.1	99	534
LF580 *	*Scopulariopsis brevicaulis*	916	*Scopulariopsis brevicaulis* strain NCPF 2177	AY083220.1	99	686
LF581	*Fusarium* sp.	504	*Fusarium* sp. NRRL 45996	GQ505760.1	99	502
LF584	*Aspergillus* sp.	543	*Aspergillus* sp. N13	GQ169453.1	99	542
LF590	*Penicillium* sp.	522	*Penicillium citreonigrum* strain Gr155	FJ904848.1	100	522
LF592	*Paecilomyces* sp.	544	Fungal endophyte sp. P1201A *Paecilomyces lilacinus* strain CG 271	EU977225.1 EU553303.1	99 98	541 516
LF594	Mycelia sterilia	531	*Fusarium* sp. FL-2010c *Fusarium acuminatum* strain NRRL 54217	HQ166535.1 HM068325.1	99 99	527 527
LF596	*Penicillium* sp.	529	*Penicillium* sp. FF24 *Penicillium canescen*s strain QLF83	FJ379805.1 FJ025212.1	100 100	529 529
LF607	*Penicillium* sp.	532	*Penicillium* sp. 17-M-1 *Penicillium sclerotiorum* strain SK6RN3M	EU076929.1 EU807940.1	99 99	523 520
LF608	*Cladosporium* sp.	495	Fungal sp. mh2981.6 *Cladosporium cladosporioide*s strain CC1	GQ996077.1 HM210839.1	100 100	495 495
LF610	*Clonostachys* sp.	528	Fungal sp. mh2053.3 *Bionectria ochroleuca* isolate Rd0801	GQ996069.1 HQ115728.1	99 99	524 524
LF626	Mycelia sterilia	588	*Trichoderma cerinum* isolate C.P.K. 3619	GU111565.1	99	586
LF627	*Aspergillus* sp.	509	*Aspergillus* sp. 4-1	HQ316558.1	100	509
LF629	Mycelia sterilia	510	*Cladosporium cladosporioide*s strain F12	HQ380766.1	99	509
LF630	*Penicillium* sp.	518	*Penicillium brevicompactum* isolate H66s1	EF634441.1	100	518
LF631	*Epicoccum nigrum*	514	*Epicoccum nigrum* strain AZ-1	DQ981396.1	99	512
LF634	*Aspergillus* sp.	581	*Aspergillus terreus* isolate UOA/HCPF 10213	GQ461911.1	99	579
LF644	*Clonostachys* sp	528	*Bionectria rossmaniae* strain CBS 211.93	AF210665.1	99	521
LF646	Mycelia sterilia	510	*Cladosporium cladosporioides* strain F12	HQ380766.1	99	509

A = anamorph; T = teleomorph; Alternaria (A) = Lewia (T); Aspergillus (A) = Petromyces (T) and Eurotium (T); Beauveria (A) = Cordyceps (T); Botrytis (A) = Botryotinia (T); Cladosporium (A) = Davidiella (T); Fusarium (A) = Gibberella (T); Clonostachys (A) = Bionectria (T); Trichoderma (A) = Hypocrea (T); Phoma = Pleurophoma (synonym); nt = nucleotides;

* phylogenetic data to LF580 are derived from the 18S rRNA gene sequence.

**Table 2.** Secondary metabolites identified in extracts of fungi isolated from the sponge *Tethya aurantium*.

**Genus**	**Strain**	**Compound**	**Reported from ^a^**	**Bioactivity ^a,b^**	**Method of dereplication**
*Alternaria*	LF177	infectopyrone	*Alternaria infectoria*, *Leptosphaeria maculans*/*Phoma lingum*		UV, MS, NMR
		phomenin A & B	*Phoma tracheiphila*, *Leptosphaeria maculans*/*Phoma lingum*, *Ercolaria funera*	phytotoxin	UV, MS, NMR
*Aspergillus*	LF627	sterigmatocystin	*Aspergillus versicolor*, *Chaetomium*	mycotoxin [32]	UV, MS
		notoamid D	*Aspergillus* sp.		UV, MS
		stephacidin A	*Aspergillus ochraceus*	cytotoxic	UV, MS, NMR
	LF547	cinereain	*Botrytis cinerea*	plant growth regulator, phytotoxin	UV, MS, NMR
		(2′*E*,4′*E*,6′*E*)-6-(1′-carboxyocta-2′,4′,6′-trien)-9-hydroxydrim-7-ene-11,12-olide	*Aspergillus ustus*		UV, MS, NMR
		(2′*E*,4′*E*,6′*E*)-6-(1′-carboxyocta-2′,4′,6′-trien)-9-hydroxydrim-7-ene-11-al	*Aspergillus ustus*		UV, MS, NMR
		compound A	hit in Scifinder [33], but no publication available		UV, MS, NMR
		compound B	no hit in database		UV, MS, NMR
	LF553	sydonic acid	*Aspergillus sydowii*	weakly antibacterial [34]	UV, MS, NMR
		hydroxysydonic acid	*Aspergillus sydowii*		UV, MS
	LF584	WIN-6 6306	*Aspergillus flavipes*	substance P antagonist, inhibition of HIV-1 integrase [35]	UV, MS
		aspochalasines	*Aspergillus flavipes* and other *Aspergillus* sp.	antibiotic, moderately cytotoxic [36]	UV, MS, NMR
*Aspergillus*/ *Petromyces*	LF491	isokotanin A–C	*Aspergillus alliaceus* and *Petromyces alliaceus*	moderate antiinsectan activities [37]	UV, MS, NMR
		14-(*N*,*N*-Dimethyl-l-leucinyloxy)paspalinine	*Aspergillus alliaceus*	potassium channel antagonist [38]	UV, MS, NMR
		nominine or a similar indoloditerpene	*Aspergillus nomius*, *Aspergillus flavus*, *Petromyces alliaceus*	insecticidal properties	UV, MS, NMR
*Phoma*	LF258	monocerin	*Helminthosporium monoceras*, *Fusarium larvarum*, *Dreschlera ravenelii*, *Exserohilum rostratum*, *Readeriella mirabilis*	antifungal, insecticidal and phytotoxic properties	UV, MS
		intermediate in the bio-synthesis of monocerin	*Dreschlera ravenelii*		UV, MS, NMR
		evernin- or isoeverninaldehyde	*Guignardia laricina*	weak phytotoxin	UV, MS, NMR
*Epicoccum*	LF552	epicoccamide	*Epicoccum purparescens* and other *Epicoccum* sp., *Aurelia aurita*		UV, MS
		orevactaene	*Epicoccum nigrum*	binding inhibitor of HIV-1 rev protein to Rev response element (RRE)	UV, MS
*Eurotium*	LF526	echinulin	*Eurotium repens*, *Aspergillus amstelodami*, *Aspergillus echinulatus*, *Aspergillus glaucus*	experimentally hepatic and pulmonary effects	UV, MS
		neoechinulines	*Aspergillus amstelodami*	antioxidative activity	UV, MS
		auroglaucines and flavoglaucine	*Aspergillus* and *Eurotium* spp.	mycotoxin, shows antineo-plastic properties [39]	UV, MS
*Fusarium*	LF236	equisetin	*Fusarium equiseti* and *Fusarium heterosporum*	antibacterial activity, inhi-bition of HIV-1 integrase	UV, MS, NMR
	LF238	equisetin	*Fusarium equiseti* and *Fusarium heterosporum*	antibacterial activity, inhi-bition of HIV-1 integrase	UV, MS, NMR
		fusarins	*Fusarium moniliforme*	mutagenic [40]	UV, MS
	LF594	enniatine	various *Fusarium* sp.	ionophore, insecticidal, ACAT inhibition, GABA receptor binding	UV, MS
*Paecilomyces*	LF592	leucinostatins	*Paecilomyces lilacinus* and other *Paecilomyces* sp.	active against Gram-positive bacteria and fungi	UV, MS
*Penicillium*	LF066	compound C	no hit in database		UV, MS, NMR
		meleagrin	*Penicillium meleagrinum* and *Penicillium chrysogenum*	structurally similar to tremorgenic mycotoxins	UV, MS, NMR
		roquefortin C	*Penicillium roquefortii* and other *Penicillium* sp.	neurotoxin	UV, MS, NMR
		sorbifuranones A–C	*Penicillium chrysogenum* [31]		UV, MS, NMR
		2′,3′-dihydrosorbicillin	*Penicillium notatum*, *Verticillium intertextum*	weakly antibacterial [41]	UV, MS
		bisvertonolone	*Penicillium chrysogenum*, *Verticillium intertextum*, *Acremonium strictum*, *Trichoderma longibrachiatum*	β-1,6glucan biosynthesis inhibitor, antioxidative, inducer of hyphal malformation in fungi	UV, MS
		ergochromes	*Aspergillus ochraceus*, *Claviceps purpurea*, *Aspergillus aculeatus*, *Gliocladium* sp., *Penicillium oxalicum*, *Phoma terrestris*, *Pyrenochaeta terrestris*	teratogenic effects	UV, MS
	LF259	mycophenolic acid	*Penicillium brevicompactum* and other *Penicillium* sp.	antineoplastig, antiviral immunosuppressant properties, useful in treating psoriasis and leishmaniasis,	UV, MS
	LF590	citreoviridins	*Penicillium citreoviride*, *Penicillium toxicarium*, *Penicillium ochrosalmoneum*, *Aspergillus terreus*	neurotoxic	UV, MS, NMR
		territrem B	*Penicillium* sp. and *Aspergillus terreus*	inhibitor of acetylcholinesterase	UV, MS
	LF607	sclerotiorin	*Penicillium sclerotiorum* and *Penicillium multicolor*	inhibits cholesterin ester transfer protein activity	UV, MS
		sclerotioramine			UV, MS, NMR
		compound D	no hit in database		UV, MS, NMR
*Penicillium*	LF596	griseofulvin	*Penicillium griseofulvum* and other *Penicillium* sp.	antifungal, possible human carcinogen	UV, MS, NMR
		tryptoquivalin	*Aspergillus clavatus*	tremorgenic toxin	UV, MS
		nortryptoquivalin	*Aspergillus clavatus* and *Aspergillus fumigatus*	tremorgenic toxin	UV, MS
		fiscalins A and C	*Neosartorya fischeri*	substance P inhibitor, neurokinin binding inhibitor	UV, MS, NMR
*Scopulariopsis*	LF580	scopularide A and B	*Scopulariopsis brevicaulis*	antiproliferative [25,26]	UV, MS, NMR
*Clonostachys*	LF254	T-988B	*Tilachlidium* sp.	cytotoxic	UV, MS, NMR
		bionectin B	*Bionectria byssicola*	antibacterial (MRSA)	UV, MS, NMR
		verticillin C	*Verticillium* sp.	antibiotic	UV, MS

a According to the Dictionary of Natural Products [42] if not stated otherwise;

b blank cells indicate that no entry concerning bioactivity in the Dictionary of Natural Products was available and no report on bioactivity was found.

**Table 3.** NMR spectroscopic data of cillifuranone (**1**) in methanol-*d*4 (500 MHz).

**Cillifuranone (1)**				
**Position**	**δ_C_, mult.**	**δ_H_, (*J* in Hz)**	**COSY**	**HMBC**
1				
2	76.3, CH_2_	4.67, s	6	3, 5, 6, 7
3	201.7, C			
4	112.6, C			
5	196.7, C			
6	31.6, CH_2_	2.76, t (7.5)	2, 7	4, 5, 7, 8
7	20.9, CH_2_	1.76, sext. (7.5)	6, 8	5, 6, 8
8	14.0, CH_3_	1.03, t (7.5)	7	6, 7
9	133.0, CH	7.32, d (16.0)	10	2, 3, 4, 5, 10, 11
10	118.5, CH	6.83, d (16.0)	9	3, 4, 5, 9, 11
11	170.9, C			
